# First person – Madeleine Clark and Antonela Losada

**DOI:** 10.1242/bio.062980

**Published:** 2026-10-30

## Abstract

First Person is a series of interviews with the first authors of a selection of papers published in Biology Open, helping researchers promote themselves alongside their papers. Madeleine Clark and Antonela Losada are co-first authors on ‘Nuclear blebs are composed of variable chromatin states and display increased transcription initiation relative to elongation’, published in BiO. Madeleine and Antonela are both lab managers in the lab of Andrew Stephens at University of Massachusetts Amherst, Amherst, MA, USA, investigating the formation and drivers of nuclear blebs, a type of abnormal nuclear morphology and hallmark of disease.

**Figure:**
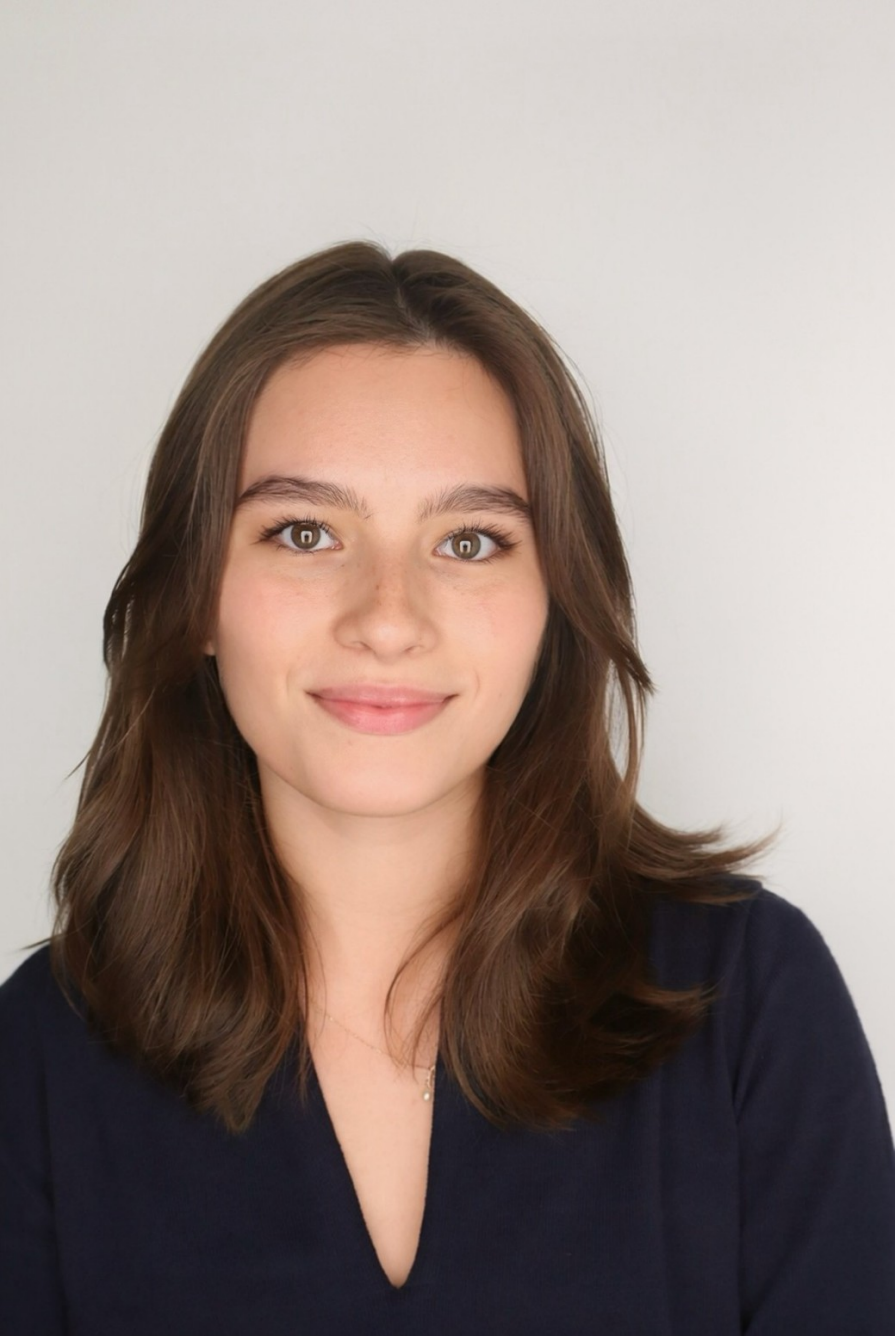
Madeleine Clark

**Describe your scientific journey and your current research focus**

We joined the Stephens lab as undergraduate students at the University of Massachusetts Amherst. Our lab is focused on investigating a type of abnormal nuclear morphology called nuclear blebbing, which hallmarks disease. We have each carried out independent projects in addition to our collaboration on this publication. Antonela investigates the mechanism behind inhibition of bromodomain and extra-terminal motif (BET) proteins and how it affects nuclear blebbing. Madeleine investigates how mechanotransduction, specifically key mechanosensor yes-associated protein (YAP), contributes to nuclear blebbing and dysfunction through modulation of actin contraction. We are both currently pursuing our Master's degrees in molecular and cellular biology at University of Massachusetts Amherst while we wrap up our independent projects and serve as lab managers for the Stephens lab.

**How would you explain the main finding of your paper?**

We care about nuclear blebs because they are a hallmark of human diseases such as advanced ageing, heart disease, muscular dystrophy and many cancers. Understanding the formation and characteristics of nuclear blebs contributes to our understanding of disease. The nucleus houses DNA, which is organized into different categories: euchromatin and heterochromatin. Euchromatin is DNA that can be read (transcribed) to become mRNA, the template for protein synthesis, while heterochromatin provides structural support to the nucleus. We found that chromatin types were variable, and that decreased DNA remains the best hallmark of a nuclear bleb. We also investigated RNA polymerase II, the key protein for transcription. This protein becomes phosphorylated (activated) at different sites during transcription. During transcription initiation it becomes phosphorylated at Serine 5 and during elongation it becomes phosphorylated at Serine 2. We found that transcription initiation was consistently increased in the nuclear bleb compared to nuclear elongation, which contributes to our understanding of what characterizes a nuclear bleb.

Our findings contribute to the field's understanding of what composes a nuclear bleb and create questions about the events that precede formation of the nuclear bleb

**What are the potential implications of this finding for your field of research?**

Our findings contribute to the field's understanding of what composes a nuclear bleb and create questions about the events that precede formation of the nuclear bleb. We now know that, in addition to decreased DNA, all blebs have increased transcription initiation relative to elongation.

**Figure:**
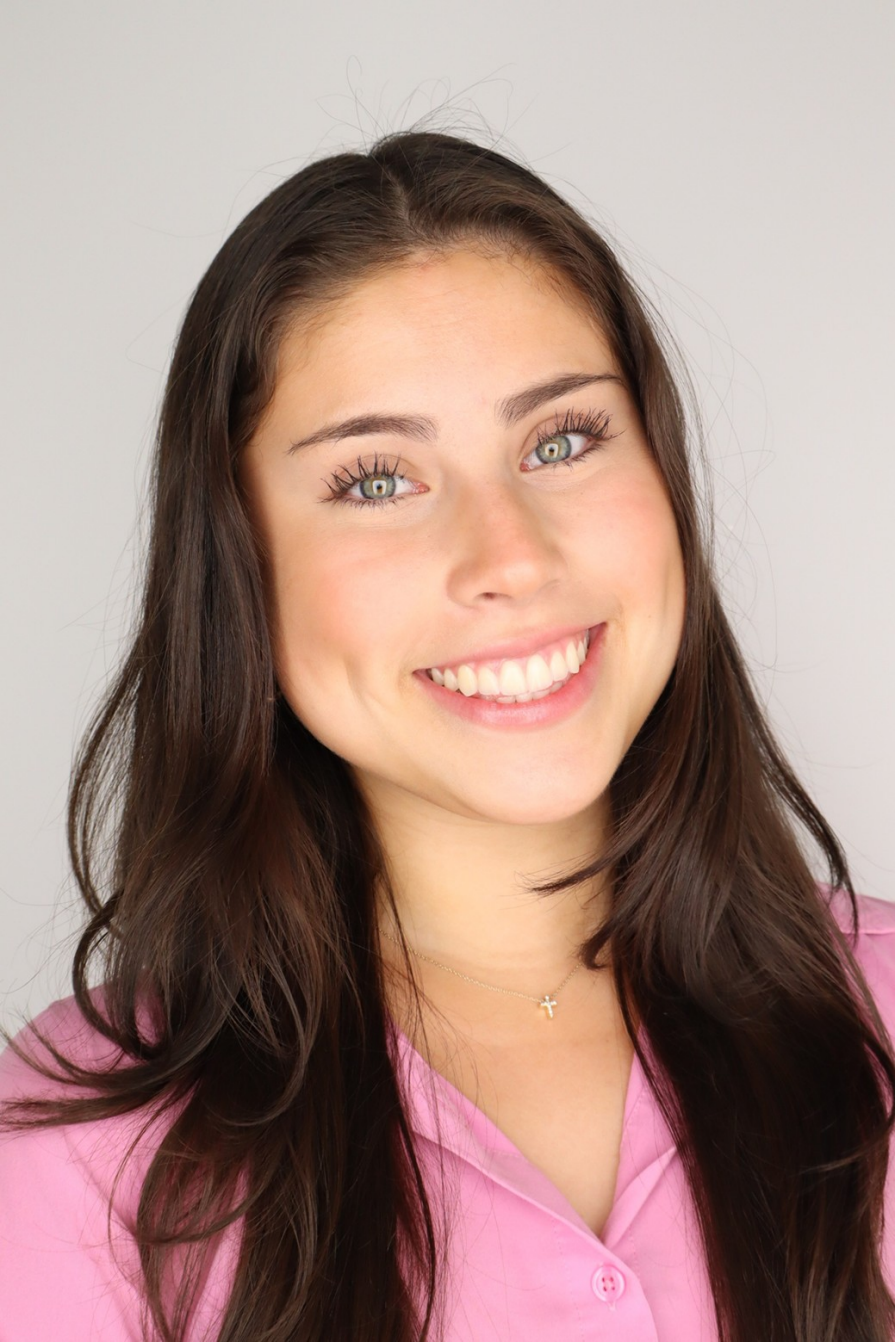
Antonela Losada

**Figure:**
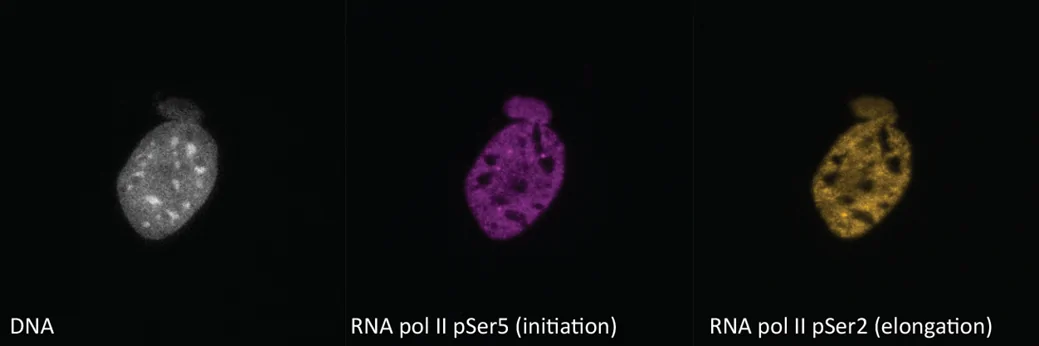
Nuclear bleb showing a clear increase in transcription initiation (magenta) compared to elongation (yellow).

**Which part of this research project was the most rewarding? (For example, this could be a new collaboration, making the key discovery, the first time the main protocol worked or presenting the results for the first time at a conference)**

The most rewarding part of this research project was seeing it all come together. This project was mainly done during a semester's long class so even though we had to work under a time crunch, collaborating with other people not in our lab was enjoyable. We got to share our research with other students and then bring the project back to lab to wrap it up.

**What do you enjoy most about being an early-career researcher?**

We enjoy the learning process and how exponential our learning and growth can be in short time periods. We can look back on where we were a couple years ago and recognize the incredible progress we have had as researchers. We also enjoy being able to work closely with our lab members and mentors to foster a collaborative environment.

**What's next for you?**

We are both hoping to finish our Master's degrees in molecular and cellular biology and graduate in May. We are also both hoping to publish our second first-author papers for each of our independent projects before we graduate. In December, we will both be presenting our data at the American Society for Cell Biology (ASCB) conference in San Diego. Madeleine hopes to pursue a PhD in molecular and cellular biology after obtaining her Master's degree.
